# Chronic Disease Burden and Physical Activity Profile in Older Adults: A Compositional Data Analysis

**DOI:** 10.1093/geroni/igaf122.2732

**Published:** 2025-12-31

**Authors:** Ethan Ouimet, Angeles Tepper, Emily Erlenbach, Stephanie Voss, Edward McAuley, Neha Gothe

**Affiliations:** Northeastern University of Bouve College of Health Sciences, Boston, Massachusetts, United States; Northeastern University, Boston, Massachusetts, United States; EXOS, South San Francisco, California, United States; Mass General Brigham, Boston, Massachusetts, United States; University of Illinois Urbana-Champaign, Urbana, Illinois, United States; Northeastern University, Boston, Massachusetts, United States

## Abstract

Understanding how physical activity (PA) levels differ by chronic disease (CD) status is essential for designing interventions to promote health. This study examined baseline differences in PA levels among older adults with varying chronic disease burdens. Participants (N = 142, mean age=64.2) were categorized into three groups: No CDs (N = 43), one CD (N = 42), or ≥two CDs (N = 57), with conditions such as diabetes and hypertension. PA profiles (sedentary time, light PA (LPA), moderate-to-vigorous (MVPA)) were assessed via accelerometry. Compositional data analysis was used to derive isometric log-ratio (ILR) coordinates representing PA distribution. Group differences were examined using linear mixed models. Participants with ≥two CDs had higher ILR1 values (Sedentary vs. Active) than those with no CDs (p = 0.032), indicating more sedentary behavior. Participants with ≥two CDs also had higher ILR2 values (LPA vs. MVPA, p = 0.006), suggesting a lower proportion of MVPA relative to LPA. Age was positively associated with both ILR1 and ILR2 (p = 0.021 and p = 0.012), meaning older participants engaged in more sedentary time and less MVPA. Women had significantly higher ILR2 values than men (p < 0.001), reflecting more LPA relative to MVPA. BMI was higher in those with ≥two CDs (p = 0.048). Sleep quality and age did not significantly differ across groups. Given these findings, older adults with multiple chronic diseases who are likely obese, may face additional barriers to PA, making a focus on increasing LPA along with MVPA a more feasible approach. PA interventions could target LPA to reduce sedentary time, with attention to strategies that support women and those with obesity.

